# A Picture Is Worth… Both Spelling and Sound

**DOI:** 10.3389/fpsyg.2018.01490

**Published:** 2018-08-17

**Authors:** Donna Coch

**Affiliations:** Reading Brains Lab, Department of Education, Dartmouth College, Hanover, NH, United States

**Keywords:** rhyme, orthography, phonology, pictures, event-related potentials, lexical processing

## Abstract

In an event-related potential (ERP) study using picture stimuli, we explored whether spelling information is co-activated with sound information even when neither type of information is explicitly provided. Pairs of picture stimuli presented in a rhyming paradigm were varied by both phonology (the two images in a pair had either rhyming, e.g., *boat* and *goat*, or non-rhyming, e.g., *boat* and *cane*, labels) and orthography (rhyming image pairs had labels that were either spelled the same, e.g., *boat* and *goat*, or not spelled the same, e.g., *brain* and *cane*). Electrophysiological picture rhyming (sound) effects were evident in terms of both N400/N450 and late effect amplitude: Non-rhyming images elicited more negative waves than rhyming images. Remarkably, the magnitude of the late ERP rhyming effect was modulated by spelling – even though words were neither explicitly seen nor heard during the task. Moreover, both the N400/N450 and late rhyming effects in the spelled-the-same (orthographically matched) condition were larger in the group with higher scores (by median split) on a standardized measure of sound awareness. Overall, the findings show concomitant meaning (semantic), sound (phonological), and spelling (orthographic) activation for picture processing in a rhyming paradigm, especially in young adults with better reading skills. Not outwardly lexical but nonetheless modulated by reading skill, electrophysiological picture rhyming effects may be useful for exploring co-activation in children with dyslexia.

## Introduction

The Lexical Quality Hypothesis (e.g., Perfetti and Hart, 2002; Perfetti, 2007) posits that the high-quality word representations that underlie fluent reading are characterized by well-integrated, automatically retrieved orthographic (spelling), phonological (sound), and semantic (meaning) information. Consistent with this, previous psycholinguistic studies have shown that both orthographic and phonological codes are accessed in young adults during both reading (e.g., Tanenhaus et al., 1980; Pexman et al., 2002) and listening (e.g., Seidenberg and Tanenhaus, 1979; Tanenhaus et al., 1980; Ziegler et al., 2004; Chéreau et al., 2007) tasks. For example, Seidenberg and Tanenhaus (1979) found that, whether a cue word was presented auditorily or visually, participants were faster to detect orthographically similar (e.g., *pie-tie*) than orthographically dissimilar (e.g., *rye-tie*) rhymes in a subsequent list of auditory words. Ziegler et al. (2004) found orthographic consistency effects on spoken word recognition in lexical decision, rime detection, and auditory naming tasks, with the strongest effects in the first and the weakest in the last. Using an auditory priming task indexing activation of prelexical representations, Chéreau et al. (2007, p. 341) also reported orthographic influence on speech perception, concluding “mandatory orthographic activation during spoken word recognition.” Indeed, the overall pattern of findings for lexical items in such psycholinguistic studies is consistent with connectivity between spelling and sound information when one or the other is provided, consonant with word processing models instantiating interactive activation of orthography and phonology in reading (e.g., McClelland and Rumelhart, 1981).

But what if no lexical information is provided? The third component in the Lexical Quality Hypothesis is semantics (e.g., Perfetti and Hart, 2002; Perfetti, 2007). What if only semantic information is provided, as in the case of picture presentation? The use of picture stimuli might address the three-way integration proposed in the Lexical Quality Hypothesis, absent the use of lexical stimuli. This could be particularly useful with populations for whom lexical stimuli are perceived as difficult and anxiety-provoking, such as children with dyslexia; indeed, this possibility motivated the present study. Can conjoint activation of spelling and sound with meaning be assessed when no lexical information is explicitly provided? Here, we used event-related potentials (ERPs) to investigate whether orthographic processing is co-activated with phonological processing when no lexical information (neither spelling nor sound) is provided, in a picture rhyming paradigm.

Event-related potential studies using prime-target pairs of stimuli and a rhyme judgment task have consistently reported a rhyming effect: Non-rhyming targets (e.g., *moose-chair*) elicit a more negative N400/N450 than rhyming targets (e.g., *moose-juice*), for both visual (e.g., Rugg, 1984b; Grossi et al., 2001; Khateb et al., 2007; Coch et al., 2008b, 2011) and auditory (e.g., Praamstra and Stegeman, 1993; McPherson et al., 1998; Radeau et al., 1998; Dumay et al., 2001; Coch et al., 2002, 2005; Perrin and García-Larrea, 2003; Wagensveld et al., 2012) linguistic stimuli. That this effect is elicited by word, pseudoword, and single letter stimuli suggests that it may be at least partially independent of semantics (e.g., Rugg, 1984a; Rugg and Barrett, 1987; Coch et al., 2008a). Thought to be primarily a phonological priming effect, such that rhyming targets that phonologically match primes require less processing (having been primed) than non-rhyming targets that phonologically mismatch primes (e.g., Rugg and Barrett, 1987; Praamstra and Stegeman, 1993; Coch et al., 2008b), this effect can nonetheless be modulated by orthography in studies with written word stimuli (e.g., Polich et al., 1983; Kramer and Donchin, 1987; Rugg and Barrett, 1987; Weber-Fox et al., 2003). For example, Weber-Fox et al. (2003) found that the amplitude of the ERP rhyming effect in adults was modulated by both phonology (typical larger N400/N450 to non-rhyming than rhyming targets) and orthographic congruency in terms of match (e.g., *thrown-own*) or mismatch (e.g., *cone-own*) between the spellings of the rime units of the prime and target words in a pair. Thus, the N400/N450 rhyming effect can provide a simultaneous index of orthographic and phonological processing of lexical items.

Critically, all of these studies have directly presented either orthographic or phonological lexical information to participants. Arguably, providing neither type of cue offers a more rigorous test of co-activation of spelling and sound with meaning. A handful of studies have shown that picture stimuli can elicit ERP rhyming effects (e.g., Barrett and Rugg, 1990; Perez-Abalo et al., 1994; McPherson et al., 1996; Wu and Thierry, 2011): both the typical N400/N450 effect and a subsequent effect extending into a later (500–700 ms) time window (McPherson et al., 1996). Pictures provide neither orthographic nor phonological information explicitly, but present the opportunity to manipulate both phonology (the names of the pictures in a pair can either rhyme, e.g., *boat* and *goat*, or not rhyme, e.g., *boat* and *cane*) and, in languages with a deep orthography such as English, orthography (the spellings of the labels for the pictures in a rhyming pair can either match, e.g., *boat* and *goat*, or mismatch, e.g., *brain* and *cane*).

Within the literature involving spoken word stimuli, there is debate about whether the effect of co-activation of spelling with sound is automatic or strategic and task-based (e.g., Damian and Bowers, 2010; Pattamadilok et al., 2011, 2014a,b; Petrova et al., 2011; Yoncheva et al., 2013). For example, Yoncheva et al. (2013) found that rhyme effects depend at least in part on attending to phonology, which in turn may activate orthography, calling into question the automaticity of orthographic effects, but Pattamadilok et al. (2014a) reported orthographic effects in an unattended spoken word oddball paradigm that varied the spelling and sound congruence between standard and deviant rimes. Some evidence suggests that a more salient orthographic manipulation induces sensitivity to and strategic use of orthographic information (e.g., Damian and Bowers, 2010, p. 108), whereas other evidence indicates a robust orthographic effect across stimulus frequencies and tasks that would seem to preclude strategic influence (e.g., Petrova et al., 2011). In particular, “metalinguistic” or “metaphonological” tasks, such as rhyme judgment, have been related to more strategic use of orthography, whereas more “on-line” or lexicosemantic tasks, such as lexical decision, have been associated with automatic processing. For example, Pattamadilok et al. (2011) reported early (175–250 ms) and late (375–750 ms) effects of orthography in an auditory rhyme judgment task in which lexical items were presented in isolation, but no effects within the 300–350 ms time window associated with orthographic effects during lexicosemantic tasks. They concluded that, in “tasks that focus on a word’s meaning, … orthographic knowledge seems to contribute to lexical access, whereas in tasks that require explicit phonological analysis, it seems to affect more peripheral processes, such as segmentation and decision” (p. 121). The present study was not designed to address this debate specifically, but rather to investigate whether pictures could be used to index orthographic and phonological co-activation in a rhyming paradigm. Given the motivation of future use with special populations, a paradigm that indexes either automatic co-activation during early processing or more controlled co-activation during late processing (or both) could be useful. However, the design of the study does provide an interesting test of these ideas: The task is metaphonological (and therefore, according to this argument, should elicit more strategic processing), but the task also focuses on meaning, as each picture stimulus must first be semantically identified (and therefore should elicit more automatic processing).

In summary, in this study, we investigated whether orthographic processing is co-activated with phonological processing in fluent, young adult readers – even when neither phonological nor orthographic lexical information is explicitly provided. Rather, the entry point was the third leg of the stool of the Lexical Quality Hypothesis: semantics (Perfetti and Hart, 2002). This afforded lexical activation in an outwardly non-lexical (i.e., image-based) task. Although previous ERP rhyming studies with picture stimuli have used cartoon drawings (e.g., Barrett and Rugg, 1990; McPherson et al., 1996), we predicted that our real-picture stimuli would elicit both a typical N400/N450 rhyming effect and the subsequent, later rhyming effect. Given reports of modulation of the N400/N450 rhyming effect by orthographic congruence in rhyming studies with written word stimuli (e.g., Weber-Fox et al., 2003) and the claims of the Lexical Quality Hypothesis (Perfetti and Hart, 2002), we further predicted that the N400/N450 picture rhyming effect would be larger for rhyming picture pairs with similarly spelled labels than dissimilarly spelled labels, consistent with dual effects of both orthographic and phonological priming. If co-activation during this task is more controlled or strategic, post lexical access, we would expect effects of orthography in the later time window. Finally, we explored associations among the rhyming effects and standardized measures of reading-related skills to determine if the ERP effects were larger for better readers, as the Lexical Quality Hypothesis suggests that better readers have more tightly integrated, precise lexical representations (e.g., Perfetti and Hart, 2002; Perfetti, 2007).

## Materials and Methods

### Participants

The final sample included 80 (40 female) participants aged 18;0 to 24;4 years (mean 20;1, *SD* 1;5). All participants were right-handed (Oldfield, 1971), monolingual native English speakers who did not self-report fluency in any other language. Also by self-report, participants had normal hearing and no history of language or reading disorders (although two reported remedial speech therapy when young children). All had normal or corrected-to-normal vision (20/30 or better) as tested with a standard Snellen chart. All were volunteers paid $20 for their participation; some participants also earned credit for Education course assignments. An additional 10 participants did not meet the 85% correct criterion on the post-test (see below) and were not included in analyses.

### Standardized Behavioral Testing

Select subtests from the Woodcock-Johnson III Tests of Achievement (WJ-III, normed to age 90+, Woodcock et al., 2003) were administered: Letter-Word Identification assessed single-word reading skill (median reliability age 5–19: 0.91, adult: 0.94); Picture Vocabulary provided a measure of expressive vocabulary (naming given pictures, median reliability age 5–19: 0.77; adult: 0.90); Spelling (median reliability age 5–19: 0.89; adult: 0.95) and Spelling of Sounds (a non-word spelling task, median reliability age 6–19: 0.74; adult: 0.82) measured orthographic knowledge; and Word Attack (reading aloud non-words, median reliability age 5–19 and adult: 0.87) and Sound Awareness (a combination of spoken word rhyming, deletion, substitution, and reversal tasks, median reliability age 5–19: 0.81; adult: 0.86) measured phonological knowledge. Scores on the Letter-Word Identification and Word Attack subtests comprise the Basic Reading Cluster (median reliability age 5–19: 0.93; adult: 0.95) and scores on the Word Attack and Spelling of Sounds subtests comprise the Phoneme/Grapheme Knowledge Cluster (median reliability age 5–19: 0.89; adult: 0.90). Finally, the Memory for Digits subtest of the Comprehensive Test of Phonological Processing served as a measure of short-term memory (CTOPP, normed to age 24;11, Wagner et al., 1999).

### ERP Task Stimuli

Initial lists of orthographically matched (e.g., *boat-goat*) and orthographically mismatched (e.g., *brain-cane*) single-syllable rhyming word pairs that could be represented by images were generated through brainstorming and web and print searches. Words composing the pairs were not in the same semantic category so as to reduce the possibility of semantic priming. Real, full-color pictures representing each word were identified using Google Images. These pictures were cropped to 1 × 1 in. with 240 pixels/in. resolution in Adobe Photoshop CS. In an iterative process, these images were subjected to cloze testing: Ten participants (none of whom participated in the actual experiment) were asked to “*label* the following pictures. What is the first word that comes to mind when you see the picture? What is the best name for the picture?” Only pictures with 80% or greater cloze scores were maintained in the final stimulus set. Responses that included the critical rhyme portion of the word were considered correct (e.g., *telephone* was a correct response for a picture intended to be labeled as *phone*). Based on this cloze procedure, average name agreement across all pictures included in the final stimulus set used in the experiment was 93.8% (*SD* = 7.5). Average name agreement was not significantly different for items in the orthographically matched (94.0%, *SD* = 7.3) and orthographically mismatched (93.6%, *SD* = 7.7) conditions, *t*(154) = 0.32, *p* = 0.75.

In addition to the pictures in the orthographically matched and mismatched conditions being labeled equally accurately in cloze pretesting, the words that the pictures represented were balanced across a number of linguistic variables based on metrics from the MCWord (Medler and Binder, 2005) and MRC Psycholinguistic (Wilson, 1988) databases (see **Table 1**). *t*-Tests confirmed that the names of the pictures in the orthographically matched and mismatched conditions did not differ in terms of number of letters, *p* = 1.0; number of phonemes, *p* = 0.16; orthographic neighborhood size, *p* = 0.12; word frequency based on the Kučera and Francis, *p* = 0.43, CELEX, *p* = 0.48, or Brown, *p* = 0.33, corpora; constrained bigram, *p* = 0.12, or trigram, *p* = 0.10, frequency; or unconstrained bigram, *p* = 0.36, or trigram, *p* = 0.13, frequency. In addition, the words in the two conditions did not differ on measures of imageability, *p* = 0.56, concreteness, *p* = 0.35, or familiarity, *p* = 0.94.

**Table 1:** Summary of stimulus word characteristics (pictures were shown, not words) for targets by orthographic condition (matching or mismatching orthography between the rimes for prime and target image labels) [mean, (SD)].

Condition	# Letters	# Phonemes	Ortho. N	K-F freq.	CELEX freq.	Brown freq.	Constrained bigram	Constrained trigram	Unconstrained bigram	Unconstrained trigram	Imageability	Concreteness	Familiarity
Match	4.2 (0.7)	3.3 (0.5)	10.3 (5.5)	63.7 (214.6)	65.2 (231.9)	19.8 (77.4)	1708.3 (1230.7)	299.9 (383.7)	18085.5 (10867.5)	2182.4 (3319.7)	587.2 (44.6)	593.6 (41.1)	538.7 (54.1)
Mismatch	4.2 (0.8)	3.2 (0.7)	8.9 (5.8)	42.5 (65.4)	45.5 (71.8)	8.1 (9.2)	1420.5 (1085.7)	212.6 (258.5)	19706.2 (11109.9)	1552.2 (1493.2)	582.5 (43.8)	585.9 (49.7)	537.9 (60.3)


*See Section “Materials and Methods” for details; metrics from Wilson (1988) and Medler and Binder (2005).*

The final stimulus set consisted of 39 pairs of orthographically matched rhyming pictures and 39 pairs of orthographically mismatched rhyming pictures. Within each rhyming pair, which picture would be the prime (presented first in the pair) and which picture would be the target (presented second in the pair) was determined randomly; *t*-tests indicated no differences between the words representing pictures designated as primes and those designated as targets on any of the linguistic variables listed above (all *p*s > 0.41). Within each orthographic condition, non-rhyming pairs were created by pseudorandomly re-pairing a previously rhyming target picture with a prime picture with which its name did not rhyme; constraints included not matching final coda consonants (orthographically or phonologically) or vowel pairs (orthographically or phonologically), and avoiding onset alliteration and semantic relatedness. For example, *goat* from the rhyming pair *boat-goat* was re-paired with the prime *crown* to create the non-rhyming pair *crown-goat*. Thus, each prime picture was seen twice (once preceding a rhyming target and once preceding a non-rhyming target) and each target picture was seen twice (once as a rhyme and once as a non-rhyme); this afforded comparison of the ERPs to the exact same image as a rhyming and a non-rhyming target in the rhyming manipulation. Picture pairs were separated randomly into a List 1 (including 20 orthographically matched rhyming picture pairs, 19 orthographically mismatched rhyming picture pairs, and 39 non-rhyming pairs) and List 2 (including 19 orthographically matched rhyming picture pairs, 20 orthographically mismatched rhyming picture pairs, and 39 non-rhyming pairs) such that each picture occurred only once in each list. The picture pairs comprising each list were presented in pseudorandom order such that no more than 3 rhyming or non-rhyming pairs occurred in a row, no more than 3 orthographically matched or mismatched pairs occurred in a row, and no target of one pair was semantically or phonologically related to the prime of the subsequent pair. All participants viewed both lists, with order of list presentation counterbalanced across participants. Please see **Supplementary Materials** for the full stimulus lists.

### Procedure

This study was approved by the Institutional Review Board at Dartmouth College, the Committee for Protection of Human Subjects. Participants were given an overview of the procedures and any questions were addressed before they signed a consent form. Standardized behavioral testing (see above), which took about 30 min, was conducted in a quiet room prior to participation in the ERP task. For electroencephalogram (EEG) recording, participants were fitted with an elastic cap (Electro-Cap International, Eaton, Ohio) with active electrodes including Fz, Cz, Pz, FP1/2, F7/8, FT7/8, F3/4, FC5/6, C3/4, C5/6, T3/4, CT5/6, P3/4, T5/6, TO1/2, and O1/2. Midline (Fz, Cz, Pz) and frontopolar (FP1/2) sites were not included in statistical analyses, but are shown in the voltage map plots. Mastoid electrodes were used for reference; on-line recordings were referenced to the right mastoid and recordings were re-referenced to averaged mastoids in the final data averaging. Electrodes located below the right eye and at the outer canthi of the left and right eyes were used to identify blinks (in conjunction with recordings from FP1/2) and horizontal eye movements, respectively. Mastoid and scalp electrode impedances were maintained below 5 KΩ, and impedances for the electrodes near the eyes below 10 KΩ. Once electrode preparation was complete, participants were seated in a comfortable chair in a sound-attenuating and electrically shielded booth for the ERP task. EEG was amplified with SA Instrumentation bioamplifiers (bandpass 0.01 to 100 Hz) and digitized on-line (sampling rate 4 ms). ERPs were time-locked to the onset of each target picture.

The picture stimuli in the ERP task were presented using Presentation software (Neurobehavioral Systems) at the center of a 19-in. LCD monitor approximately 66 in. in front of each participant, on a black background. The images subtended about 1.9° of horizontal visual angle, minimizing the need for scanning eye movements. The sequence of events began with a red asterisk at the center of the screen, to which participants needed to press either one of two buttons on a hand-held response device to advance to the presentation of the prime stimulus. Upon pressing a button, the prime picture was presented for 500 ms, followed by a gray fixation cross for 900 ms, the target picture for 500 ms, a blank screen for 500 ms, and a blue question mark that appeared for a maximum of 3 s. Participants were asked to press one button to indicate a rhyme decision and another to indicate a non-rhyme decision as quickly as possible after the onset of the question mark; rhyme/non-rhyme response hand was counterbalanced across participants. The question mark disappeared with the press of a response button (or when 3 s had elapsed), to be replaced by the red asterisk to begin the next trial. The session was self-paced in that only a button press by the participant would advance from the asterisk to a stimulus pair. On average, participants were slower to complete the first list presented (mean = 7.8 min, *SD* = 3.3) than the second list (mean = 6.7 min, *SD* = 2.2), *t*(79) = 2.66, *p* = 0.01; however, since presentation order of Lists 1 and 2 was counterbalanced, time to complete each list was not significantly different, *p* = 0.98. A brief practice session (8 pairs, including no pictures used in the actual experiment) preceded the experimental session.

In a post-test following the ERP task, participants were asked to label each picture (using the label that came to mind during the ERP task) by typing in a name for each picture in an Excel file. A criterion for inclusion in further analyses was at least 85% correct on this post-test.

### Data Analysis

Off-line, ERPs to target pictures were averaged for each subject at each electrode site over a 1000 ms epoch, using a 200 ms pre-stimulus-onset baseline. Only trials to which participants responded correctly and within the allotted time were included in the ERP averages. Trials contaminated by eye movements, blinks, or electrical noise were not included in analyses. Standard artifact rejection parameters were initially employed: Blinks and eye movements were detected through a “peak-to-peak amplitude” function, and trials were rejected if the amplitude value between the maximum and minimum data points in the specified time window was larger or smaller than an *a priori* established threshold. Data were subsequently analyzed on an individual basis for artifact rejection as needed, by modifying the threshold if blinks or eye movements were still visible in the individual average data after the standard, automatized procedure. The average number of trials included were: rhyming targets, mean 59.8 (*SD* = 8.0), and non-rhyming targets, mean 67.5 (*SD* = 9.1). More non-rhyming than rhyming target trials were included in analyses, *t*(79) = 10.12, *p* = 0.001. Within rhyming targets, the average number of trials included was greater for orthographically matching (mean = 31.3, *SD* = 4.6) than orthographically mismatching (mean = 28.4, *SD* = 4.3) targets, *t*(79) = 7.12, *p* = 0.001. Within non-rhyming targets, the average number of trials included was also slightly greater for pictures in the orthographically matching set (mean = 34.0, *SD* = 4.6) than in the orthographically mismatching set (mean = 33.2, *SD* = 5.4), *t*(79) = 2.11, *p* = 0.038.

As planned, consistent with previous work and visual inspection of both individual and grand average data here, mean amplitude of the N400/N450 was measured in the 300–500 ms epoch and the late effect was measured in the 500–700 ms time window. In addition, given the suggestion of an effect in the individual and grand average waveforms, we analyzed the mean amplitude of the N280, an anteriorly distributed component previously shown to be sensitive to repetition in a picture paradigm (Wang et al., 1998), in the 200–300 ms time window. To address the first research question concerning ERP rhyming effects for real-picture stimuli, the mean amplitude data from ERPs to targets were analyzed in each of the three time windows in initial ANOVAs with within-subjects factors rhyme condition (rhyming, non-rhyming), anterior/posterior [6 levels: frontal (F7/8, F3/4), fronto-temporal (FT7/8, FC5/6), temporal (T3/4, C5/6), central (CT5/6, C3/4), temporoparietal (T5/6, P3/4), and occipital (TO1/2, O1/2)], hemisphere (left/right), and lateral/medial. To better visualize the effects, difference waves (waves resulting from the subtraction of the rhyming target ERPs from the non-rhyming target ERPs) were created and plotted as topographical voltage maps using a spherical spline interpolation (Perrin et al., 1989) to interpolate the potential on the surface of an idealized, spherical head based on the mean amplitude measures from the grand average difference waves at each electrode location in each of the three time windows of interest. Difference waves were created for the overall rhyming effect, for the rhyming effect for orthographically matching targets [waves resulting from the subtraction of the orthographically matching rhyming target ERPs from the orthographically matching non-rhyming target ERPs (that is, the same subset of target images when in rhyming and non-rhyming contexts)], and for the rhyming effect for orthographically mismatching targets (waves resulting from the subtraction of the orthographically mismatching rhyming target ERPs from the orthographically mismatching non-rhyming target ERPs). Subsequently, to address the second research question concerning modulation of rhyming effects by orthography, ANOVAs with mean amplitude data from the difference waves were performed in each of the three time windows, allowing for direct comparison of the rhyming effects for the orthographically matching and mismatching targets. For these analyses, within-subjects factors included orthographic condition (match, mismatch), anterior/posterior, hemisphere, and lateral/medial. The Greenhouse-Geisser correction was applied to all within-subjects measures with more than one degree of freedom, and corrected *p*-values are reported. Partial eta squared ($\eta_{p}^{2}$) values are reported as estimates of effect size. All results were considered significant at the 0.05 level.

Finally, exploratory, planned analyses by group were conducted to investigate possible effects related to individual differences. Groups were created by median split of standard scores on the Sound Awareness subtest (the subtest most directly related to the ERP rhyme task), and were compared in terms of the sizes of the ERP rhyming effects (from the difference waves, overall and for orthographically matching and mismatching targets separately) at the sites where each effect was maximal (N280: right hemisphere, anterior, medial sites F4, FC6, and C6; N400/N450: right hemisphere, frontocentral, medial sites F4, FC6, C6, and C4; late effect: right hemisphere, posterior, medial sites C3, C4, P3, P4, O1, and O2).

## Results

### Behavioral Tasks

#### Standardized Behavioral Tests

For the most part, participant scores were average or above, although there was variability. Results, in terms of raw, percentile, and standard scores, are summarized in **Table 2**.

**Table 2:** Summary of standardized behavioral test results [mean, (SD)], overall and for the subgroups created by median split of standard scores on the Sound Awareness subtest of the WJ-III.

Group and Score Type	WJ-III Letter-Word ID	WJ-III Spelling	WJ-III Word Attack	WJ-III Picture Vocabulary	WJ-III Spelling of Sounds	WJ-III Sound Awareness	WJ-III Cluster Basic Reading	WJ-III Cluster Phon/Graph Knowledge	CTOPP Memory for Digits
*Overall*									
Raw score	72.5 (2.4)	53.9 (2.9)	28.9 (2.2)	35.3 (3.7)	37.0 (2.3)	42.2 (1.7)	–	–	17.9 (2.5)
Percentile rank	60.3 (18.4)	74.0 (18.7)	47.3 (23.8)	51.7 (25.3)	62.9 (24.6)	44.3 (25.7)	55.3 (20.6)	54.5 (23.3)	76.9 (21.1)
Standard score	104.5 (8.1)	111.5 (9.8)	99.0 (10.3)	100.8 (12.6)	107.4 (13.9)	97.8 (11.7)	102.5 (9.3)	102.2 (10.5)	12.7 (2.2)
*Lower Sound Awareness*									
Raw score	71.6 (2.5)	53.3 (2.9)	28.1 (2.4)	34.3 (3.7)	36.4 (2.1)	40.9 (1.1)	–	–	17.6 (2.9)
Percentile rank	51.5 (16.7)	69.2 (19.2)	37.3 (21.5)	43.8 (23.4)	53.9 (23.2)	22.7 (8.2)	44.5 (18.0)	43.2 (20.3)	73.0 (22.8)
Standard score	100.8 (7.0)	109.0 (9.6)	94.7 (9.6)	96.5 (11.4)	103.0 (12.5)	88.3 (4.3)	98.1 (8.4)	97.6 (9.5)	12.3 (2.4)
*Higher Sound Awareness*									
Raw score	73.5 (1.9)	54.6 (2.7)	29.8 (1.6)	36.3 (3.4)	37.6 (2.3)	43.5 (1.1)	–	–	18.1 (2.1)
Percentile rank	69.1 (15.7)	78.9 (17.0)	57.3 (21.9)	59.6 (24.9)	71.9 (22.8)	65.9 (17.9)	66.1 (17.2)	65.8 (20.6)	80.8 (18.7)
Standard score	108.3 (7.4)^***^	114.0 (9.3)^*^	103.3 (9.3)^***^	105.1 (12.3)^**^	111.9 (13.9)^**^	107.3 (8.6)^***^	106.8 (8.2)^***^	106.9 (9.5)^***^	13.1 (2.1)


*See Section “Materials and Methods” for details; from Wagner et al. (1999) and Woodcock et al. (2003). Standard score differences, by t-test, between the lower-scoring and higher-scoring Sound Awareness groups indicated in the higher-scoring row: ^∗^p ≤ 0.05; ^∗∗^p ≤ 0.01; ^∗∗∗^p ≤ 0.001.*

#### Accuracy on the ERP Task

Overall accuracy was 89.4% (*SD* = 4.7%). *t*-Tests indicated that participants were more accurate at identifying non-rhyming (raw mean = 74.4, *SD* = 2.3) than rhyming (raw mean = 65.1, *SD* = 6.0) targets, *t*(79) = 14.4, *p* < 0.001, and more accurate with orthographically matching (raw mean = 34.3, *SD* = 2.9) than mismatching (raw mean = 30.8, *SD* = 3.7) rhyming targets, *t*(79) = 10.8, *p* < 0.001.

#### Accuracy on the Post-test

On average, participants correctly identified 92.5% (*SD* = 3.5%) of the pictures at post-test, similar to the results of our cloze procedure for choosing the picture stimuli (i.e., 93.8%, *SD* = 7.5%).

### ERP Task

**FIGURE 1:**
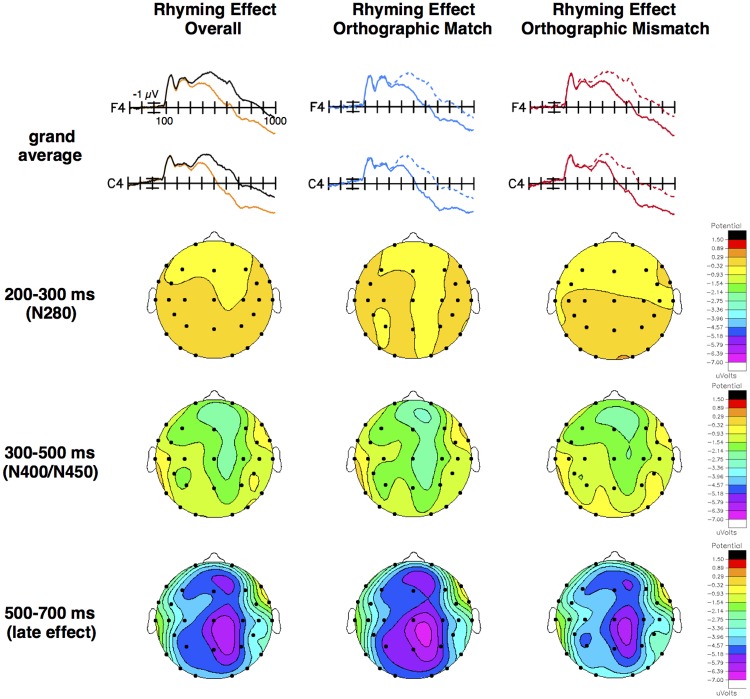
Grand average ERP waveforms and topographical voltage maps. Grand averages and voltage maps for the rhyming effect overall, and for orthographically matched (e.g., *boat* and *goat*) targets and orthographically mismatched (e.g., *brain* and *cane*) targets separately. Note that picture stimuli (no words) were shown. Top row: Grand averages at illustrative right hemisphere, medial, frontal (F4) and central (C4) sites for ERPs elicited by all non-rhyming targets (black) and all rhyming targets (orange), rhyming (solid blue) and non-rhyming (dashed blue) targets in the orthographically matched condition, and rhyming (solid red) and non-rhyming (dashed red) targets in the orthographically mismatched condition. Negative is plotted up and the calibration bar marks 1 μV. Subsequent rows: Difference waves (non-rhyming – rhyming targets) were created to directly compare the rhyming effects (see **Figure 2**), and differences (the size of the rhyming effect in μV) were plotted as voltage maps on the same scale for all three time windows and effects.

We found a typical ERP rhyming effect on N400/N450 amplitude such that non-rhyming targets (mean = -2.48 μV, *SE* = 0.29) elicited a more negative N400/N450 than rhyming targets (mean = -1.36 μV, *SE* = 0.34), particularly over right hemisphere, frontocentral, medial sites (see **Figure 1** and **Table 3**). For example, the size of the rhyming effect was smaller at the temporal, lateral, left hemisphere site T5 (mean = -0.46 μV, *SE* = 0.13) than it was at the frontal, medial, right hemisphere site F4 (mean = -1.96 μV, *SE* = 0.23), *t*(79) = 6.74, *p* < 0.001, *d* = 0.75, and smaller at the frontal, lateral, left hemisphere site F7 (mean = -0.42 μV, *SE* = 0.16) than it was at the frontocentral, medial, right hemisphere site FC6 (mean = -1.58 μV, *SE* = 0.19), *t*(79) = 6.51, *p* < 0.001, *d* = 0.73.

**Table 3:** Summary of ANOVAs for ERP mean amplitude elicited by rhyming vs. non-rhyming target pictures in grand average waveforms and the rhyming effects for orthographically matching vs. orthographically mismatching target pictures in difference waves in three time windows.

Comparison and source		200–300 ms		300-500 ms		500-700 ms	
				
	*df*	*F*	$\eta_{p}^{2}$	*F*	$\eta_{p}^{2}$	*F*	$\eta_{p}^{2}$
**Rhyming vs. Non-rhyming Targets**							
C	1, 79	3.87	0.05	66.11^***^	0.46	326.31^***^	0.81
C × H	1, 79	0.76	0.01	24.65^***^	0.24	35.47^***^	0.31
C × A/P	5, 395	1.24	0.02	4.79^***^	0.06	64.98^***^	0.45
C × L/M	1, 79	1.44	0.02	46.14^***^	0.37	215.44^***^	0.30
C × H × A/P	5, 395	0.88	0.01	6.48^***^	0.08	34.39^***^	0.30
C × H × L/M	1, 79	0.27	0.003	11.74^**^	0.13	12.25^***^	0.13
C × A/P × L/M	5, 395	1.88	0.02	21.45^***^	0.21	97.66^***^	0.55
C × H × A/P × L/M	5, 395	1.04	0.01	3.48^**^	0.04	6.63^***^	0.08
**Rhyming effects: Orthographic**							
**Match vs. Mismatch**							
C	1, 79	0.97	0.001	0.13	0.002	2.32	0.03
C × H	1, 79	0.64	0.003	0.02	0.00	1.42	0.02
C × A/P	5, 395	3.04^∧^	0.04	2.61^∧^	0.03	5.58^**^	0.07
C × L/M	1, 79	0.21	0.02	2.51	0.03	3.45^∧^	0.04
C × H × A/P	5, 395	2.46^∧^	0.03	0.63	0.008	0.65	0.008
C × H × L/M	1, 79	1.73	0.19	1.48	0.018	0.08	0.001
C × A/P × L/M	5, 395	0.27	0.003	0.35	0.004	0.79	0.01
C × H × A/P × L/M	5, 395	1.86	0.02	1.23	0.015	0.90	0.01


*C, condition; H, hemisphere; A/P, anterior/posterior; L/M, lateral/medial (see Section “Materials and Methods” for details). ^∧^p ≤ 0.1; ^∗^p ≤ 0.05; ^∗∗^p ≤ 0.01; ^∗∗∗^p ≤ 0.001.*

This pattern extended into the 500–700 ms time window, with non-rhyming targets (mean = -1.47 μV, *SE* = 0.32) eliciting a more negative late effect than rhyming targets (mean = 1.85 μV, *SE* = 0.37), particularly over right hemisphere, posterior, medial sites. For example, the size of the late rhyming effect was smaller at the frontal, lateral, left hemisphere site F7 (mean = -0.64 μV, *SE* = 0.20) than at the parietal, medial, right hemisphere site P4 (mean = -4.98 μV, *SE* = 0.25), *t*(79) = 16.81, *p* < 0.001, *d* = 1.88, and at the temporal, lateral, left hemisphere site T5 (mean = -2.68 μV, *SE* = 0.22) than at the central, medial, right hemisphere site C4 (mean = -4.80 μV, *SE* = 0.28), *t*(79) = 8.63, *p* < 0.001, *d* = 0.96.

**FIGURE 2:**
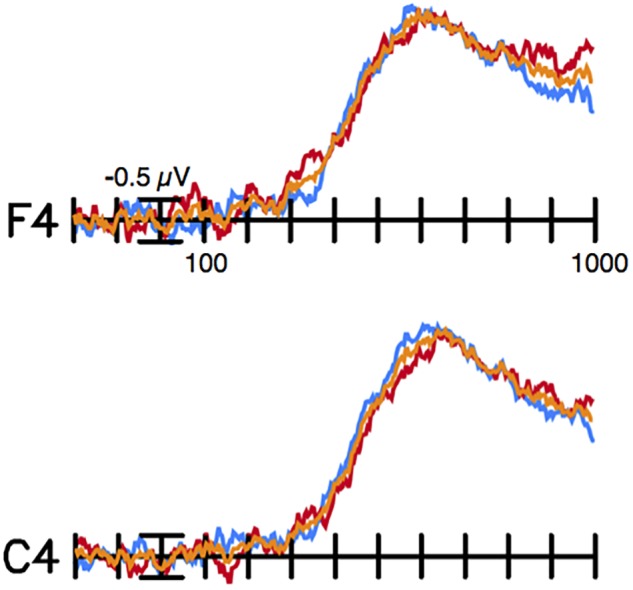
Difference waves. Difference waves created by subtracting rhyming target ERPs from non-rhyming target ERPs. The overall rhyming effect is shown in orange, the rhyming effect for picture targets in the orthographically matched condition is shown in blue, and the rhyming effect for picture targets in the orthographically mismatched condition is shown in red. Recordings from right hemisphere, medial, frontal (F4) and central (C4) sites are shown. Negative is plotted up and the calibration bar marks 0.5 μV. These same data are represented as topographical voltage maps in **Figure 1**.

In difference wave analyses investigating whether the orthographic congruence (i.e., matchedness of spelling) between primes and targets modulated these effects, there were no conventionally significant effects in either the N280 or N400/N450 time windows (see **Figures 1, 2** and **Table 3**). In the later epoch, the rhyming effect for orthographically matched targets was larger than the rhyming effect for orthographically mismatched targets, particularly at posterior sites. Follow-up analyses indicated no significant effect of orthography across the electrode sites composing the two most anterior rows (F7, F3, F4, F8, FT7, FC5, FC6, FT8), *p* = 0.33, but a larger effect for orthographically matched than mismatched targets across the sites composing the two most posterior rows (T5, P3, P4, T6, TO1, O1, O2, TO2), *t*(79) = -2.94, *p* < 0.01, *d* = 0.33.

### Standardized Test/ERP Categorical Analyses

**FIGURE 3:**
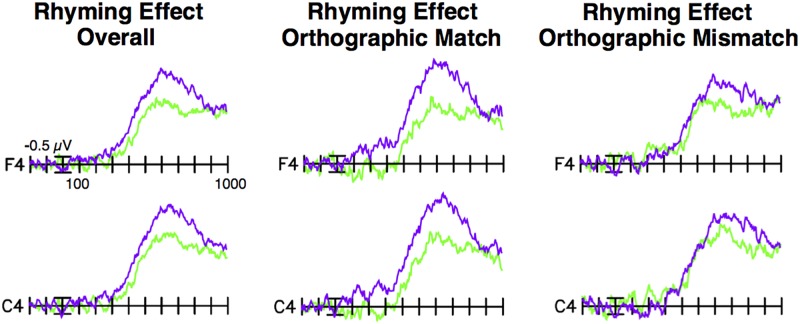
Difference waves by sound awareness group. Difference waves (non-rhyming – rhyming targets) by group based on a median split of standard scores on the WJ-III Sound Awareness subtest. Difference waves for the higher-scoring group are shown in purple, and for the lower-scoring group in green. The higher-scoring group had a larger N400/N450 rhyming effect overall, as well as larger N280, N400/N450, and late rhyming effects for orthographically matched picture targets, in comparison to the lower-scoring group.

In planned analyses exploring possible categorical effects related to individual differences, participants were divided into lower-scoring and higher-scoring groups (*n* = 40) by median split of standard scores on the Sound Awareness subtest (see **Figure 3** and **Table 2**). The median split (cut-off: 95) created significantly different lower-scoring (mean = 88.3, *SD* = 4.3) and higher-scoring (mean = 107.3, *SD* = 8.6) groups, *t*(78) = -12.43, *p* < 0.001, *d* = 2.8. (In further analyses, standard scores for these groups differed, in the expected direction, on every WJ-III administered subtest and cluster; see **Table 2**). The size of the N280 rhyming effect for orthographically matched pictures was greater for the higher-scoring group (mean = -0.92 μV, *SE* = 0.37; low: mean 0.37 μV, *SE* 0.29), *t*(78) = 2.78, *p* < 0.01, *d* = 0.62. For the N400/N450, both the size of the rhyming effect overall (high: mean = -2.09 μV, *SE* = 0.24; low: mean = -1.30 μV, *SE* = 0.30), *t*(78) = 2.02, *p* < 0.05, *d* = 0.45, and the size of the rhyming effect for orthographically matched pictures (high: mean = -2.30 μV, *SE* = 0.33; low: mean = -1.05 μV, *SE* = 0.35), *t*(78) = 2.63, *p* = 0.01, *d* = 0.59, were larger for the higher-scoring group. The magnitude of the late rhyming effect for orthographically matched pictures was also larger for the higher-scoring (mean = -5.54 μV, *SE* = 0.33) than the lower-scoring (mean = -4.30 μV, *SE* = 0.43) group, *t*(78) = 2.28, *p* < 0.05, *d* = 0.51. Thus, across all three time windows in this exploratory analysis, the pattern is consistent with greater combined phonological and orthographic priming for participants with better phonological skills.

## Discussion

A rhyming paradigm with picture stimuli requires participants to generate the phonological form of a meaningful label for each image, a task-based elicitation of phonology. If orthographic information is co-activated with meaning and phonological information within the lexicosemantic system, ERP rhyming effects in a picture rhyming paradigm should be modulated by the orthographic congruence of the spellings of the labels for the pictures in each pair. Of the three ERP picture rhyming effects measured here, only the late effect showed evidence of modulation by orthography. However, individual differences in phonological skill were associated with differences in combined orthographic and phonological priming across all three time windows.

Real-picture stimuli elicited both the typical N400/N450 and late rhyming effects previously reported in ERP rhyming studies (e.g., Rugg, 1984b; McPherson et al., 1998; Coch et al., 2002, 2008b; Wagensveld et al., 2012): Non-rhyming targets elicited more negative waves within the 300–500 and 500–700 ms epochs than rhyming targets. Moreover, for the amplitude of the late effect, there was greater priming for rhyming targets with orthography matched to primes’, suggesting that orthographic and phonological congruence worked in concert to reduce processing demands. A similar pattern was apparent in the accuracy results, with participants correctly responding “rhyme” more often for orthographically matched than orthographically mismatched rhyming picture pairs.

Given that, overall, significant modulation of the amplitude of the rhyming effects by orthography occurred only in the late (500–700 ms) time window, it would seem that co-activation of spelling and sound in this paradigm was strategic and/or task-related (e.g., Damian and Bowers, 2010; Pattamadilok et al., 2011). Some previous authors have proposed that such late effects may be related to the confidence of the rhyme/non-rhyme decision (e.g., Ackerman et al., 1994; McPherson et al., 1996). Similarly, Pattamadilok et al. (2011, p. 121) interpreted their late (375–750 ms) effect of orthography in an auditory rhyme judgment task in terms of decision-making processes. Others employing behavioral phonological priming paradigms have discussed post-lexical processes that check for congruency between prime and target (e.g., Radeau et al., 1989; Norris et al., 2002), which may be indexed by the late effect in this rhyming paradigm. This is consistent with Pattamadilok et al.’s (2011, p. 121) claim that, for tasks that require explicit phonological analysis, like rhyme judgment, orthographic knowledge does not seem to contribute to lexical access.

And yet there was other evidence suggesting that orthographic congruence may indeed contribute to lexical access – and, thus, that orthography may have both automatic and strategic influence – in this picture rhyming paradigm. In planned, exploratory analyses, the group with higher scores on the Sound Awareness subtest by median split showed a larger overall N400/N450 rhyming effect, as might be expected: Better phonological skills were associated with greater differentiation of rhyming and non-rhyming targets. But the group with higher scores on the Sound Awareness subtest also showed larger N280, N400/N450, and late rhyming effects for orthographically matched (but not mismatched) targets, as compared to the lower-scoring group. Thus, participants with better speech sound processing skills (as measured by the WJ-III Sound Awareness subtest, which involves no orthography) appeared to benefit more from phonological-orthographic congruence (in terms of less processing cost, or more priming related to the dual effects of phonology and orthography) than those with poorer speech sound processing skills, across all three time windows of measurement. Moreover, the higher-scoring Sound Awareness group also had higher standard scores on the WJ-III Letter-Word Identification, Spelling, Word Attack, Picture Vocabulary, and Spelling of Sounds subtests, as well as on the Basic Reading and Phoneme/Grapheme Knowledge Clusters, in comparison to the lower-scoring Sound Awareness group, suggesting that greater dual phonological-orthographic priming may not be specific to sound-based skills, but rather to better reading skill overall. Intriguing, this greater facilitative effect of spelling and sound congruence in the orthographic match condition for better, as compared to poorer, readers awaits replication. Although metaphonological, the picture rhyming task also focuses on meaning, as each image must be semantically identified for retrieval of its label. Typical rhyme judgment tasks tend to be “shallow” lexical processing tasks (Pattamadilok et al., 2011, p. 120); in the picture rhyming paradigm, that lexical retrieval is dependent on meaning may trigger “deeper” lexical processing, especially in more-skilled readers. Thus, this rhyming paradigm may be particularly suitable for investigating co-activation in readers with varying skill levels.

An influence of orthographic congruence that varied by group was observed across all three time windows. The N280 elicited here is similar to a right-lateralized, anterior N2 that was associated with maintenance of context information, and was largest to repeated images, in a previous study with picture stimuli (Wang et al., 1998). The repetition of both phonology and orthography in orthographically matched picture pairs may have modulated the amplitude of this component in the median split analyses. Consistent with this interpretation, in a written word study, a similar right frontal effect has been associated with orthographic processing during rhyme judgments (Rugg and Barrett, 1987). Although the timing and distribution are not perfectly matched, this early component may also be related to the PMN observed in auditory studies, elicited by mismatch between an expected and presented initial phoneme (e.g., Connolly et al., 1992, 1995; Connolly and Phillips, 1994); however, in this case, one might have expected a similar effect for orthographically mismatched pairs, which was not significant. An effect of orthographic congruence that varied by group was also apparent in the middle (N400/N450) time window, which has consistently been associated with lexical access and lexicosemantic processing at multiple levels of representation (e.g., Coch and Holcomb, 2003; Grainger and Holcomb, 2009; Laszlo and Federmeier, 2011). If processing within this epoch indexes a “form-meaning interface,” it seems reasonable that such interfacing or integration could differ with participant skill level (Grainger and Holcomb, 2009, p. 141). Finally, the same pattern was observed in the late time window typically indexing post-lexical processing (e.g., Pattamadilok et al., 2011), as discussed above.

Taken together, these findings suggest that co-activation of spelling and sound in a rhyme task – both automatic, as indexed in early time windows, and strategic, as indexed in later time windows – may not only be task-dependent (e.g., Damian and Bowers, 2010; Pattamadilok et al., 2011), but also dependent on participant skill level (cf. Pattamadilok et al., 2014b). Overall, the exploratory median split analyses based on phonological skill (and, likely, single-word reading skill more generally) suggest that neural lexical representations in fluent readers involve tightly integrated orthography and phonology, so much so that the latter does not come without the former, even under circumstances in which neither is directly provided. In particular, the congruence of spelling and sound is more strongly linked or represented in young adults with better sound awareness (and other reading) skills, consistent with better readers having more tightly integrated lexicosemantic representations (Perfetti and Hart, 2002). In turn, this is consistent with the speculation that “the impact of selective attention to phonological information in driving obligatory recruitment of orthographic information during… rhyming might be relevant to behavioral outcomes” (Yoncheva et al., 2013, p. 241), and suggests that the picture rhyming paradigm may be useful with special populations with differing behavioral skills.

## Conclusion

Our findings of orthographic modulation of phonological effects in a picture rhyming task are consistent with the automatic co-activation of meaning, sound, and spelling proposed in the Lexical Quality Hypothesis (e.g., Perfetti and Hart, 2002; Perfetti, 2007), as well as strategic use of orthographic information in a rhyme task (e.g., Pattamadilok et al., 2011). Greater combined orthographic and phonological neural priming effects in participants with better behavioral sound awareness (and basic reading) skills lent support to the notion that better readers have more integrated word representations, and may use orthography differently in this task during both early and late processing, as compared to poorer readers. In a stringent test of the hypothesis of co-activation in a paradigm that provided neither spelling nor sound information explicitly, we found evidence of concomitant semantic, phonological, and orthographic activation upon viewing a target picture. Thus, this ostensibly non-reading-related ERP picture rhyming paradigm may be useful for investigating co-activation in children with dyslexia.

## Author Contributions

DC conceptualized and designed the study, collected, analyzed, and interpreted the data, wrote the manuscript, and supervised undergraduate research assistants.

## Conflict of Interest Statement

The author declares that the research was conducted in the absence of any commercial or financial relationships that could be construed as a potential conflict of interest. The reviewer SD and handling Editor declared their shared affiliation.
