# Supplementary material for: A Picture Is Worth… Both Spelling and Sound
Source: Front Psychol. 2018 Aug 17;9:1490. doi: 10.3389/fpsyg.2018.01490 (PMC6107714; doi:10.3389/fpsyg.2018.01490)
Supplement: Supplementary file 1 [file Table_1.DOCX]

Supplementary Material

A Picture is Worth… Both Spelling and Sound

Donna Coch*

*** Correspondence:**Dr. Donna Coch
donna.coch@dartmouth.edu

# Supplementary Data: Stimulus List (pictures were shown)

**LIST 1**  **LIST 2**

veil-goose stool-pool

nose-rose tires-bowl

mouse-top shell-bell

grill-floss toast-bra

tires-pliers bread-bed

shell-fan brain-stew

chain-fork crown-goat

key-bee gum-thumb

mop-pill bride-slide

lips-chips hook-book

teeth-rake cone-swing

yarn-barn tie-whale

bow-hoe doll-hoe

pan-van mouse-house

bride-jug cross-floss

crown-clown car-king

gum-bed tree-ghost

grape-tape plum-drum

fence-beer soap-cane

boat-goat tear-beer

hook-slide hose-keg

brain-cane wig-barn

steak-tail steak-rake

juice-wheel shawl-rope

ring-swing bow-tea

seal-thumb can-fan

soap-rope rice-dice

screw-ear ring-plow

snake-peach socks-box

fire-shoe boat-clown

chess-dress snake-cake

hose-toes saw-toes

axe-bear lips-van

knees-skis nun-pig

tag-flag pear-hair

train-scale deer-ear

cow-plow snail-scale

chair-box spoon-tape

doll-ball ham-lamb

chick-pool chess-horn

corn-horn glue-door

noose-brick chick-brick

smore-door mop-top

wing-king rug-jug

dart-heart knees-fly

rug-bell noose-moose

tree-tea maid-jar

cork-house grape-bun

egg-keg screw-shoe

wig-pig juice-goose

nun-bun chain-rain

deer-jacks tag-braid

beach-moose train-vane

glue-stew clock-lock

shawl-wall nose-moon

bread-vane pan-rose

spoon-moon fire-choir

saw-bra smore-wall

tie-fly egg-skis

clock-dice corn-dress

toast-ghost grill-pill

pear-fox fence-tents

truck-duck teeth-wreath

stool-cake cow-flag

maid-braid beach-peach

snail-tents nail-heart

cone-phone axe-jacks

rocks-hair wing-phone

cross-book veil-tail

rice-lock cork-fork

sole-bowl chair-bear

car-jar truck-chips

socks-choir dart-pliers

plum-rain rocks-fox

nail-whale sole-bee

ham-wreath yarn-duck

can-drum key-ball

tear-lamb seal-wheel
